# The Assessment of Bone Regulatory Pathways, Bone Turnover, and Bone Mineral Density in Vegetarian and Omnivorous Children

**DOI:** 10.3390/nu10020183

**Published:** 2018-02-07

**Authors:** Jadwiga Ambroszkiewicz, Magdalena Chełchowska, Katarzyna Szamotulska, Grażyna Rowicka, Witold Klemarczyk, Małgorzata Strucińska, Joanna Gajewska

**Affiliations:** 1 Department of Screening and Metabolic Diagnostics, Institute of Mother and Child, Kasprzaka 17A, 01-211 Warsaw, Poland; magdalena.chelchowska@imid.med.pl (M.C.); joanna.gajewska@imid.med.pl (J.G.); 2 Department of Epidemiology and Biostatistics, Institute of Mother and Child, Kasprzaka 17A, 01-211 Warsaw, Poland; katarzyna.szamotulska@imid.med.pl; 3 Department of Nutrition, Institute of Mother and Child, Kasprzaka 17A, 01-211 Warsaw, Poland; grazyna.rowicka@imid.med.pl (G.R.); witold.klemarczyk@imid.med.pl (W.K.); malgorzata.strucinska@imid.med.pl (M.S.)

**Keywords:** bone metabolism markers, bone mineral density, vegetarian diet, prepubertal period

## Abstract

Vegetarian diets contain many beneficial properties as well as carry a risk of inadequate intakes of several nutrients important to bone health. The aim of the study was to evaluate serum levels of bone metabolism markers and to analyze the relationships between biochemical bone markers and anthropometric parameters in children on vegetarian and omnivorous diets. The study included 70 prepubertal children on a lacto-ovo-vegetarian diet and 60 omnivorous children. Body composition, bone mineral content (BMC), and bone mineral density (BMD) were assessed by dual-energy X-ray absorptiometry. Biochemical markers—bone alkaline phosphatase (BALP), C-terminal telopeptide of type I collagen (CTX-I), osteoprotegerin (OPG), nuclear factor κB ligand (RANKL), sclerostin, and Dickkopf-related protein 1 (Dkk-1)—were measured using immunoenzymatic assays. In vegetarians, we observed a significantly higher level of BALP (*p* = 0.002) and CTX-I (*p* = 0.027), and slightly lower spine BMC (*p* = 0.067) and BMD (*p* = 0.060) than in omnivores. Concentrations of OPG, RANKL, sclerostin, and Dkk-1 were comparable in both groups of children. We found that CTX-I was positively correlated with BMC, total BMD, and lumbar spine BMD in vegetarians, but not in omnivores. A well-planned vegetarian diet with proper dairy and egg intake does not lead to significantly lower bone mass; however, children following a lacto-ovo-vegetarian diet had a higher rate of bone turnover and subtle changes in bone regulatory markers. CTX-I might be an important marker for the protection of vegetarians from bone abnormalities.

## 1. Introduction

Vegetarianism has been gaining popularity among families as a lifestyle choice, since the health benefits of a vegetarian diet have been reported [1,2,3,4]. According to experts’ statements, the plant-based dietary pattern, if well balanced, can be adequate for all stages of the life cycle, including childhood [5,6,7]. Since nutrition is one of the determinants of bone health, an insufficiency of animal products decreases intake of some essential nutrients (minerals, vitamins), and may affect bone metabolism. Potential deficiencies of protein, calcium, vitamin D, and vitamin B_12_ observed in vegetarian diets are of particular concern in terms of bone health [8,9].

This is especially important in periods of childhood and adolescence, when growth and development are the most intensive [10,11]. The balance between bone formation and resorption processes is controlled by many factors that influence osteoblast and osteoclast activity and are regulated through signaling pathways, such as the complex of RANK/RANKL/OPG cytokines, consisting of receptor activator of nuclear factor κB (RANK), receptor activator of nuclear factor κB ligand (RANKL), and osteoprotegerin (OPG) [12,13,14]. An increase in the RANKL/OPG ratio is associated with an imbalance in osteoclastic/osteoblastic activity, leading to increased bone resorption and decreased bone mass [12,13].

In bone metabolism, activation of Wnt-β-catenin signaling stimulates the bone formation process by increasing osteoblast proliferation and differentiation. Its regulation is dependent on many proteins, including sclerostin and Dickkopf-related protein 1 (Dkk-1) [14,15,16,17]. Sclerostin and Dkk-1 are antagonists of canonical Wnt signaling, both binding to the low-density lipoprotein receptor-related protein 5/6 (LRP 5/6) on osteoblasts, block the signaling cascade and consequently inhibit bone formation [18]. Sclerostin is synthetized by osteocytes and decreases bone formation by inhibiting the terminal differentiation of osteoblasts and by promoting their apoptosis [19,20]. Dkk-1 is expressed not only by osteocytes but also by osteoblasts and adipocytes and is involved in the mechanism of the activation of peroxisome proliferator-activated receptor-γ (PPAR-γ), which promotes the differentiation of mesenchymal stem cells into adipocytes over osteoblasts [14,21]. Wnt-β-catenin signaling plays an important role in the regulation of bone mass, osteoblastogenesis, and osteocyte and adipocyte function.

The effect of vegetarian diets on bone status has been investigated mainly in adults. Researchers found slightly decreased or comparable bone mineral density (BMD) in lacto-ovo-vegetarians and significantly decreased BMD in vegans [22,23]. In a meta-analysis, Ho-Pham et al. [24] observed lower (by 4%) lumbar spine BMD in adult vegetarians compared with omnivores. Little is known about levels of bone turnover markers in vegetarians. Generally, similar levels of C-terminal telopeptide of type I collagen (CTX), bone alkaline phosphatase (BALP), and osteocalcin were observed in adult vegetarians on a well-planned diet [25,26]. There are only a few reports assessing bone status in children following vegetarian diets [27,28]. In our previous study, we found decreased BMD in adolescents on a vegan diet [29]. None of the studies have measured levels of markers related to regulatory pathways such as RANKL, OPG, sclerostin, and Dkk-1 in vegetarian children.

The aim of the study was to evaluate the serum levels of bone metabolism markers in prepubertal children on vegetarian and omnivorous diets and to analyze the relationships between these biochemical markers and bone mineral density in subjects following different kind of diets.

## 2. Methods

### 2.1. Subjects

Our study group consisted of 70 prepubertal children (age range 5–10 years) following a vegetarian diet, specifically the lacto-ovo-vegetarian diet, in which meat, fish are not consumed, but poultry, but eggs, milk, and dairy products are consumed. We included in the study the maximum possible number of prepubertal vegetarian children who followed a lacto-ovo-vegetarian diet from birth and had a densitometry as well as a biochemical examination done simultaneously. The inclusion criteria regarding vegetarians were as follows: being on a lacto-ovo-vegetarian diet from birth, in the prepubertal period, generally healthy (without development and nutrition disorders, Body Mass Index (BMI) *z*-score between −1 and +1), more than 2 h/week of physical exercise. The exclusion criteria were as follows: not in the prepubertal period, history of low birth weight, gastrointestinal diseases accompanied by malabsorption, history of chronic renal failure, chronic infection, chronic drug consumption or drug use that negatively affected bone metabolism. These rigorous selection criteria were applied in order to minimalize interference from factors that could affect normal bone metabolism.

The control group included 60 children (age range 5–10 years) following a traditional omnivorous diet which included meat, fish and poultry. Children were recruited between January 2014 and April 2017 from a group of consecutive patients attending the Department of Nutrition at the Institute of Mother and Child in Warsaw. Pubertal stage was assessed according to the Tanner criteria. The protocol of this study was approved by the Ethics Committee of the Institute of Mother and Child (number 13/2012) and conducted according to the principles of the Declaration of Helsinki. Written informed consent was obtained from the children’s parents.

### 2.2. Dietary Assessment

Methods used for measuring dietary intake in the studied children was described in detail previously [30]. Before medical consultation the parents were asked to prepare a 10-day food diary for their children. They had been previously advised by a nutritionist on how to do it correctly. In the Department of Nutrition, the diary was checked by the nutritionist in the presence of the child and parent. The nutritionist asked for detailed information about the foods and drinks recorded, such as portion sizes and preparation methods, using a photo album of products and dishes. When necessary, the food diary was corrected by the nutritionist during the visit. Out of the 10 days in the record, three consecutive days (two weekdays and one weekend day) were selected. These data were entered into a nutritional software program, Dieta5^®^ (National Food and Nutrition Institute, Warsaw, Poland), for analysis, to evaluate average daily energy intake, the percentage of energy from protein, fat and carbohydrates, as well as dietary mineral and vitamin intakes in the children’s diets. The obtained results were compared (for each child for the appropriate age and sex) with the current recommendations for Polish children, according to Jarosz et al. [31].

### 2.3. Anthropometric Parameters

Height and weight measurements were performed in all children and body mass index (BMI) was calculated as body weight (kg) divided by height squared (m^2^). Fat mass, lean mass, bone mineral content (BMC), and bone mineral density (BMD) in the total body (tBMD) and at the lumbar spine (BMD L1–L4) were measured by dual-energy X-ray absorptiometry (DXA) using Lunar Prodigy (General Electric Healthcare, Madison, WI, USA). BMD *z*-scores were obtained using the pediatric reference population database, enCORE.

### 2.4. Biochemical Measurements

Blood samples were obtained between 8:00 a.m. and 10:00 a.m. after an overnight fast. Serum samples were collected and kept frozen at −20 °C until analysis (no longer than 2 months). Concentrations of bone metabolism markers were determined by commercial enzyme-linked immunosorbent assay (ELISA), according to the manufacturer’s instructions. Serum CTX-I concentrations were measured using Serum CrossLaps kits from Immunodiagnostic System (Boldon, UK). The intra-and inter-assay coefficients of variation (CVs) were 1.7–3.0% and 2.5–10.9%. Serum BALP, OPG, and sclerostin concentrations were determined using kits from Quidel (San Diego, CA, USA). The intra-and inter-assay CVs were 3.9–5.8% and 5.0–7.6% for BALP, 2.1–3.5% and 4.2–6.1% for OPG, and 3.7–4.2% and 4.2–4.8% for sclerostin, respectively. The soluble RANKL level was determined using kits from Immundiagnostik AG (Bensheim, Germany) with intra-assay CVs from 0.9% to 3.5% and inter-assay from 7.1% to 9.3%. Serum Dkk-1 level was measured using a kit from BioVendor (Brno, Czech Republic); the intra-assay and inter-assay precisions were 3.5–5.9% and 5.6–8.6%, respectively.

### 2.5. Statistical Analysis

All statistical analyses were carried out using IBM-SPSS software version 23.0 (SPSS Inc., Chicago, IL, USA). The Kolmogorov–Smirnov test was used to test the normality of the distribution of the studied parameters. For variables with normal distribution, average values were compared using the student’s *t*-test, whereas for variables with skewed distributions, significance was assessed with the Mann–Whitney test. Where appropriate, Pearson or Spearman correlation coefficients were calculated to evaluate the relationships between biochemical bone metabolism markers and bone mineral density parameters. Multivariate regression models with total BMD and lumbar spine BMD as dependent variables were assessed to examine associations with bone metabolism markers after adjustment for age and sex for vegetarian and omnivore groups. A *p*-value of <0.05 was considered statistically significant.

## 3. Results

Analyzing the children’s diets, we found that average daily energy intake in both groups of children was within recommendations, whereas the proportions of macronutrient intakes in vegetarians and omnivores were different. Compared with omnivores, vegetarians had a similar percentage of energy from fat (31.5 ± 5.4 vs. 32.8 ± 6.2, *p* = 0.318), a lower percentage of energy from protein (11.4 ± 1.9 vs. 15.6 ± 4.2, *p* < 0.001), and a higher percentage of energy from carbohydrates (57.1 ± 5.8 vs. 52.3 ± 5.9, *p* = 0.004). Dietary intake of dairy products and eggs in both studied groups of children were comparable. The median value (and 25–75% range) of dairy products in the vegetarian diet was 368.3 (143.6–555.3) g/day and in the omnivorous diet was 401.6 (243.2–641.0) g/day (*p* = 0.318). Regarding eggs, the median value was 30.8 (5.6–49.8) g/day in the vegetarian and 28.4 (10.5–45.6) g/day in the omnivorous diet (*p* = 0.556). Also, intake of protein from dairy and eggs was similar in the two studied groups (intake of protein from dairy: 10.9 g/day (3.2–15.3 g/day) in vegetarians and 12.3 g/day (6.8–16.1 g/day) in omnivores (*p* = 0.331); intake of protein from eggs: 3.4 (0.7–5.4) mg/day in vegetarians and 3.2 (1.2–5.3) mg/day in omnivores (*p* = 0.465)). In both groups of children, we observed sufficient intake of phosphorus (866 ± 330 vs. 930 ± 330 mg/day, *p* = 0.377), but insufficient dietary intake of calcium (548 ± 315 vs. 596 ± 222 mg/day, *p* = 0.410) and vitamin D (1.74 (0.82–4.32) vs. 2.01 (1.13–4.64) µg/day, *p* = 0.575). Only 55% of vegetarians and 64% of omnivores followed the recommended daily intake for calcium and 38% of vegetarians and 45% of omnivores for vitamin D. Additionally, vegetarian children had significantly lower intakes of vitamin B_12_ compared with omnivores: 1.36 (0.84–1.76) vs. 2.51 (1.85–3.72) µg/day, *p* < 0.001.

Both groups of children were similar in terms of age, weight, height, BMI, and body composition (Table 1). Mean values of body composition parameters (fat mass, lean mass, the ratio of fat/lean mass) and total and spine BMC as well and total and lumbar spine BMD were comparable in vegetarians and omnivores.

Regarding bone metabolism markers, we observed higher levels of BALP and CTX-I in vegetarians than in omnivores (*p* = 0.002 and *p* = 0.027, respectively) and comparable concentrations of OPG, RANKL, sclerostin, and Dkk-1 (Table 2). We also measured serum concentrations of 25-hydroxyvitamin D (25-OH D) and parathormone (PTH) in both groups of studied children and observed that the mean value of 25-OH D was similar (24.9 ± 10.5 ng/mL in vegetarians and 26.3 ± 8.5 ng/mL in omnivores, *p* = 0.476); however, the median value of PTH was higher in vegetarians: 35.7 (27.9–49.8) pg/mL than in omnivores: 29.9 (20.0–35.9) pg/mL (*p* = 0.041).

**Figure 1:**
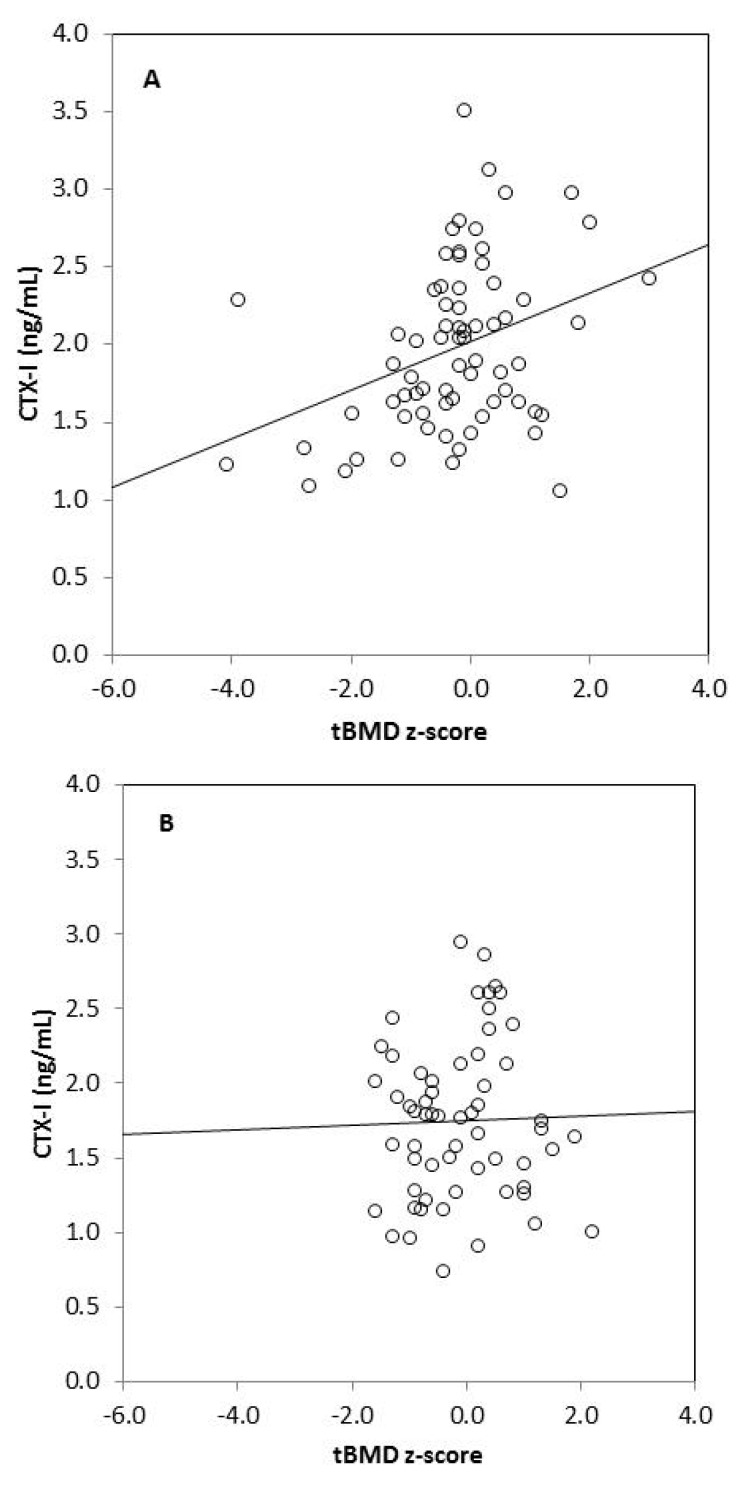

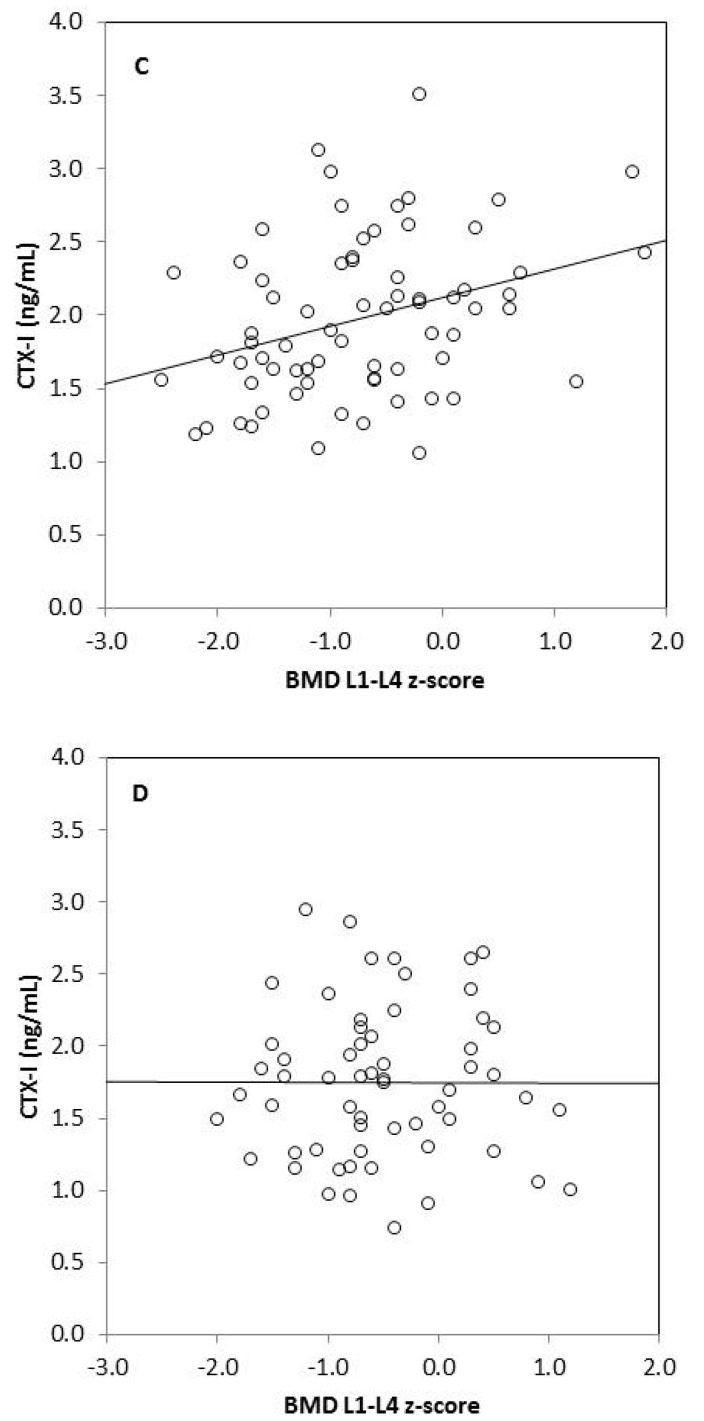
Correlation between serum CTX-I level and tBMD *z*-score in vegetarians (*r* = 0.349, *p* = 0.003) (**A**); between CTX-I and tBMD *z*-score in omnivores (*r* = 0.026, *p* = 0.846) (**B**); between CTX-I and BMD L1–L4 *z*-score in vegetarians (*r* = 0.330, *p* = 0.005) (**C**) and between CTX-I and BMD L1–L4 in omnivores (*r* = 0.004, *p* = 0.977) (**D**).

We found strong significant positive correlations between tBMC, spine BMC, tBMD, BMD L1–L4, and anthropometric variables (body mass, height, BMI, fat mass, lean mass) in omnivores and vegetarians, except between tBMD and fat mass in vegetarians (Table 3). Generally, there was no correlation between biochemical bone metabolism markers and BMC as well as BMD in the omnivorous group, except for a weak negative correlation between tBMC and RANKL (*r* = −0.255, *p* = 0.049). In the vegetarian group, we observed positive significant correlations between CTX-I and tBMC, spine BMC, tBMD, as well as BMD L1–L4. Moreover, spine BMC was positively correlated with BALP and negatively with OPG in this group of children. We also found significant associations between bone metabolism markers: CTX-I and BALP (*r* = 0.255, *p* = 0.033), CTX-I and sclerostin (*r* = 0.273, *p* = 0.022), and between BALP and OPG (*r* = 0.305, *p* = 0.010) in vegetarian children, but not in the omnivorous group (data not shown). Associations between serum CTX-I and tBMD or CTX-I and lumbar spine BMD also remained significant in children on a vegetarian diet (*p* = 0.024 and *p* = 0.002, respectively) and non-significant in children following an omnivorous diet (*p* = 0.347 and *p* = 0.699, respectively), after adjustment for age and sex in linear regression models (Table 4). Additionally, we observed that serum CTX-I significantly correlated with tBMD *z*-scores and with BMD L1–L4 *z*-scores in vegetarians (*r* = 0.349, *p* = 0.003 and *r* = 0.330, *p* = 0.005, respectively) (Figure 1). These relationships were not found in omnivores (*r* = 0.026, *p* = 0.846 and *r* = 0.004, *p* = 0.977, respectively).

## 4. Discussion

In the present study, we observed that there were no significant differences in body composition, bone mineral content and bone mineral density between prepubertal children on a vegetarian diet with proper dairy and egg intake and children on a traditional omnivorous diet. However, we found that children following a lacto-ovo-vegetarian diet had a higher rate of bone turnover and subtle changes in bone regulatory markers. Analyzing the correlations, the values of BMCs and BMDs were highly positively associated with weight, height, BMI, fat mass, and lean mass in both studied groups of children, except for a correlation between total BMD and fat mass in vegetarian children. The fact that lumbar spine BMD was more compromised is not surprising, because it reflects the density of trabecular bone, which shows very active bone metabolism. In constrast, total body BMD represents cortical bone, which is also influenced by other factors, such as mechanical loading. Childhood and adolescence represent periods when girls and boys can achieve optimal bone mass for the prevention of osteopenia and osteoporosis during adulthood. Optimization of bone mass is determined by genetic factors but also by modifiable environmental factors, such as physical activity and diet [32]. Although bone mineral density is considered an important, precise, and valid measurement, BMD does not reflect the dynamic changes the bone has undergone. Thus, the use of biochemical markers is important for understanding the balance between the bone formation and bone resorption processes [33]. Studies on adults suggest that vegetarians and omnivores had similar serum levels of bone formation and resorption markers, without evidence of increased bone turnover [25,26,34]. The observed significantly higher (by about 10%) levels of BALP and CTX-I in the vegetarian children than in omnivores, suggest an increased rate of bone turnover in these subjects. 

This is the first study describing bone regulatory markers, such as RANKL and OPG, in vegetarians. We did not find significant differences in the serum concentrations of these parameters in vegetarian and omnivorous children. The RANK/RANKL/OPG cytokine system is identified as a critical regulator of osteoclastogenesis and thus, bone resorption [13]. Higher PTH, slightly increased levels of RANKL together with higher CTX-I in our children on a vegetarian diet may indicate higher bone resorption in these subjects, but the direct mechanism of these changes is not clear. Moreover, it is unclear whether serum concentrations of OPG and RANKL reflect their activity in the bone microenvironment.

We determined, also for the first time in vegetarians, serum levels of regulatory markers related to the Wnt signaling pathway, and found similar sclerostin and Dkk-1 concentrations in vegetarians and omnivores. The Wnt/beta-catenin pathway plays a critical role in bone formation and bone mass acquisition [17]. Sclerostin and Dkk-1 are powerful negative regulators of osteoblastogenesis. An increase in their levels results in reduced osteoblast differentiation and thus decreased bone formation [14,35,36,37]. Although the mean values of these markers were comparable in both groups of children, sclerostin concentrations were slightly lower in vegetarians. As sclerostin is recognized to be an important inhibitor of bone formation by the Wnt-signaling pathway, its lower levels may be an adaptive response, an attempt to protect the skeleton from unrestrained bone formation. Our results suggest that other mechanisms, not solely associated with RANKL/OPG or sclerostin and Dkk-1, might be involved in explaining the increased bone turnover and slightly lower bone mineral density in vegetarian children.

Analyzing correlations between biochemical markers and anthropometric parameters, we found that serum CTX-I was significantly correlated with BMC in the total body and in the spine and with BMD (total as well as lumbar spine) in vegetarians. Additionally, in this group of children, we observed a weak correlation between spine BMC and serum BALP as well as OPG levels. It is worth noting that we did not find such associations in children on an omnivorous diet. Moreover, only in vegetarians, we found significant associations between bone metabolism markers: CTX-I and BALP, CTX-I and sclerostin, and between BALP and OPG. 

In light of these findings, CTX-I is a biochemical marker that correlated with BMC and BMD z-scores in vegetarian children. It is intriguing that this resorption parameter was positively associated with bone mineral density. In the study conducted by Kemp et al. [38], the authors found that CTX-I was positively associated with bone size, as reflected by periosteal circumference (PC), in contrast to inverse associations with cortical BMD related to bone remodeling in a large cohort of healthy adolescents. They hypothesized that a positive association between CTX-I and PC reflects a casual pathway whereby increased bone resorption leads to an increase in bone size. Periods of rapid growth, such as childhood, are associated with marked increases in bone resorption and formation markers. Bone resorption plays a primary role in modeling and remodeling, such that periosteal expansion occurs secondary to increased resorption. It is suggested that periosteal expansion represents part of an overall response intended to retain bone strength. An alternative explanation is that it may be the case that changes in serum bone metabolism markers do not accurately reflect changes occurring at the tissue level.

It is known that proper nutrition is very important for bone mineralization [39]. Our vegetarian children met the recommendations regarding macronutrients; however, their dietary intake of protein was lower by 27% compared with omnivores, expressed as a percentage of energy. We observed that the lacto-ovo-vegetarian and omnivorous children had similar intakes of milk, dairy products and eggs as well as intake of protein from these products in their diets. We suggest that the lack of differences in the bone parameters of both studied groups of children may be due to the similar intakes of dairy products and eggs in their diets. Moreover, our vegetarians follow a lacto-ovo-vegetarian diet, which includes milk, dairy products, and eggs. Their dietary intake of calcium and vitamin D was comparable with non-vegetarians; however, intake of vitamin B_12_ was significantly lower than in omnivores. It is known that vitamin B_12_ is a high risk nutrient for vegetarians, especially vegans [10]. Low vitamin B_12_ may lead to an increase in homocysteine (Hcy), which was observed in adults on long-term vegetarian or vegan diets. Recently, hyperhomocysteinemia has been linked to be a potential risk factor for osteoporosis [40]. This suggestion is derived from the observation of high bone turnover and low BMD in patients with increased homocysteine levels [41,42]. It was found that increased Hcy stimulates human osteoclast activity, suggesting its role in bone resorption [43]. Saito et al. [44] reported that homocysteine disturbs enzymatic collagen crosslinking by the inhibition of lysyl oxidase. Researchers reported that in adults on long-term vegetarian diets, the prevalence of low vitamin B_12_ status was 11% in omnivores, 77% in lacto-ovo-vegetarians, and 92% in vegans [45]. However, in our previous study, we observed a normal level of homocysteine in prepubertal children following a vegetarian diet [46]. The absence of osteoporosis in vegetarians can be explained, at least partially, by the alkalizing effect of fruits and vegetables and higher intake of magnesium and vitamins K and C, which may have a protective effect on bone status [9].

The limitations of our study included a relatively small sample, which lacks sufficient power to detect moderate associations with statistical significance. Our analyses were limited to prepubertal children who had undergone densitometry and biochemical analyses at the same time. However, groups of children on vegetarian and omnivorous diets were compared in terms of ethnicity (all participants were Caucasian), age, sex, weight, height, and BMI. All children followed a lacto-ovo-vegetarian diet from birth. Secondly, we detected only BALP and CTX among bone turnover markers. However, for the first time, we assessed serum concentrations of regulatory markers, such as OPG, RANKL, sclerostin and Dkk-1, which provide information about the balance between osteoclast and osteoblast activity. Furthermore, some RANKL (about 10%) are not detected in the circulation, thus the interpretation ratio of OPG/RANKL was limited due to the relatively high percentage of participants with undetectable RANKL. Finally, we did not consider the physical activity of the studied children in the analysis, which is an important factor affecting bone health. However, they were healthy children who took part in sport activities at school and outside school. 

## 5. Conclusions

Our results show that a well-planned vegetarian diet with proper dairy and egg intake does not lead to significantly lower bone mass. However, children following a lacto-ovo-vegetarian diet had a higher rate of bone turnover and subtle changes in bone regulatory markers. It is not excluded that CTX-I is the biomarker that better reflects changes in bone status in vegetarians. Its positive correlations with bone mineral content and bone mineral density might be important for the protection of vegetarians from bone abnormalities. Precisely how the protective factors interact with potential deficiencies in the vegetarian diet remains unclear.

## Figures and Tables

**Table 1:** Clinical and anthropometric data in vegetarian and omnivorous children.

	Vegetarian Children (*n* = 70)	Omnivorous Children (*n* = 60)	*p*
Age (years) ^a^	6.56 ± 1.8	6.88 ± 1.5	0.287
Weight (kg) ^a^	22.16 ± 5.53	23.26 ± 5.79	0.275
Height (cm) ^a^	119.2 ± 11.5	122.3 ± 10.8	0.114
BMI (kg/m^2^) ^a^	15.4 ± 1.4	15.5 ± 1.6	0.760
BMI *z*-score ^a^	−0.352 ± 0.645	−0.302 ± 0.616	0.674
Fat (kg) ^b^	3.34 (2.79–4.79)	3.76 (3.05–5.86)	0.126
Lean (kg) ^b^	16.57 (13.97–18.08)	16.99 (13.58–20.66)	0.501
Fat/lean ^b^	0.24 (0.18–0.29)	0.28 (0.20–0.33)	0.098
Total BMC (g) ^a^	729 ± 226	768 ± 237	0.341
Spine BMC (g) ^a^	57.5 ± 18.7	63.0 ± 18.1	0.093
Total BMD (g/cm^2^) ^a^	0.784 ± 0.068	0.799 ± 0.080	0.700
Total BMD *z*-score ^a^	−0.26 ± 1.20	−0.11 ± 0.89	0.433
Lumbar spine BMD L1–L4 (g/cm^2^) ^a^	0.617 ± 0.083	0.645 ± 0.083	0.060
Lumbar spine BMD L1–L4 z-score ^a^	−0.73 ± 0.91	−0.51 ± 0.75	0.114

Data are presented as ^a^ mean values ± standard deviation (SD), ^b^ median values and interquartile ranges (1Q–3Q), BMI—body mass index, BMC—bone mineral content, BMD—bone mineral density, BMD L1–L4—lumbar spine L1–L4 bone mineral density.

**Table 2:** Serum concentrations of biochemical bone metabolism markers in vegetarian and omnivorous children.

	Vegetarian Children (*n* = 70)	Omnivorous Children (*n* = 60)	*p*
BALP (U/L) ^a^	130.7 ± 39.9	108.4 ± 37.1	0.002
CTX-I (ng/mL) ^a^	1.976 ± 0.538	1.749 ± 0.526	0.027
OPG (pmol/L) ^a^	4.27 ± 1.08	4.29 ± 1.19	0.918
RANKL (pmol/L) ^b^	1635 (619–3726)	1418 (716–3184)	0.790
OPG/RANKL ratio ^b^	0.22 (0.10–0.67)	0.23 (0.12–0.65)	0.996
Sclerostin (ng/mL) ^a^	0.436 ± 0.133	0.457 ± 0.110	0.346
Dkk-1 (ng/mL) ^a^	2.694 ± 0.950	2.661 ± 0.896	0.848

Data are presented as ^a^ mean values ± standard deviation (SD), ^b^ median values and interquartile ranges (1Q–3Q); BALP—bone alkaline phosphatase, CTX-I—carboxyterminal telopeptide of collagen type I, OPG—osteoprotegerin, RANKL—receptor activator of nuclear factor κB ligand, Dkk-1—Dickkopf-related protein 1.

**Table 3:** Correlations between bone mineral content as well bone mineral density and biochemical or anthropometric parameters in children on vegetarian and omnivorous diets.

	tBMC		Spine BMC		tBMD		BMD L1–L4	
	*r*	*p*	*r*	*p*	*r*	*p*	*r*	*p*
Vegetarians								
BALP	0.218	0.070	0.138	0.256	0.270	0.024	0.230	0.056
CTX-I	0.282	0.018	0.375	0.001	0.272	0.023	0.343	0.004
OPG	−0.263	0.028	−0.257	0.031	−0.049	0.687	−0.224	0.062
RANKL	0.013	0.913	0.065	0.594	0.007	0.955	0.150	0.215
OPG/sRANKL ratio	0.010	0.938	−0.068	0.589	0.009	0.943	−0.125	0.320
Sclerostin	0.027	0.823	−0.002	0.990	−0.064	0.596	0.092	0.450
Dkk-1	−0.085	0.484	−0.059	0.630	0.069	0.571	−0.052	0.668
Weight	0.881	<0.001	0.783	<0.001	0.529	<0.001	0.635	<0.001
Height	0.884	<0.001	0.750	<0.001	0.508	<0.001	0.628	<0.001
BMI	0.423	<0.001	0.438	<0.001	0.306	0.010	0.315	0.008
Fat mass	0.358	0.002	0.425	<0.001	0.111	0.359	0.419	<0.001
Lean mass	0.902	<0.001	0.809	<0.001	0.737	<0.001	0.552	<0.001
Omnivores								
BALP	0.067	0.610	0.090	0.496	0.040	0.760	0.009	0.944
CTX-I	0.174	0.183	0.066	0.616	0.066	0.615	0.099	0.452
OPG	0.087	0.507	0.080	0.541	0.092	0.484	0.071	0.592
RANKL	−0.255	0.049	−0.200	0.125	−0.144	0.388	0.003	0.984
OPG/sRANKL ratio	0.221	0.095	0.179	0.178	0.099	0.461	−0.013	0.925
Sclerostin	0.080	0.545	0.118	0.369	0.107	0.414	0.222	0.088
Dkk-1	0.035	0.813	0.061	0.681	0.178	0.227	−0.070	0.636
Weight	0.881	<0.001	0.882	<0.001	0.651	<0.001	0.676	<0.001
Height	0.840	<0.001	0.823	<0.001	0.630	<0.001	0.689	<0.001
BMI	0.633	<0.0001	0.647	<0.001	0.479	<0.001	0.438	<0.001
Fat mass	0.524	<0.001	0.635	<0.001	0.380	0.003	0.577	<0.001
Lean mass	0.929	<0.001	0.828	<0.001	0.813	<0.001	0.650	<0.001

BALP—bone alkaline phosphatase, CTX-I—carboxyterminal telopeptide of collagen type I, OPG—osteoprotegerin, RANKL—receptor activator of nuclear factor κB ligand, Dkk-1—Dickkopf-related protein 1, BMI—body mass index.

**Table 4:** The relationship between total body BMD and lumbar spine BMD and serum CTX-I adjusted for age and sex in vegetarian (*n* = 70) and omnivorous (*n* = 60) children.

	Vegetarian Children			Omnivorous Children		
	B	95% CI	*p*	B	95% CI	*p*
Dependent variable: total BMD						
CTX-I	0.032	0.004–0.059	0.024	−0.016	−0.049–0.017	0.347
Dependent variable: lumbar spine BMD						
CTX-I	0.049	0.019–0.079	0.002	−0.006	−0.039–0.026	0.699

BMD—bone mineral density, CTX-I—carboxyterminal telopeptide of collagen type I.
